# A Rapid and Highly Effective Protocol for Sporophyte Proliferation of *Drynaria roosii* and the Potential Regulation Mechanism by Plant Growth Regulators

**DOI:** 10.3390/plants15162543

**Published:** 2026-08-21

**Authors:** Yanlei Han, Yan Ren, Ye Cao, Siyuan Zhen, Xiwen Li, Ye Wang

**Affiliations:** 1 Jiangxi Province Key Laboratory of Sustainable Utilization of Traditional Chinese Medicine Resources, Institute of Traditional Chinese Medicine Health Industry, China Academy of Chinese Medical Sciences, Nanchang 330115, China; hyacinth426534@163.com (Y.H.); wwwgpg@163.com (Y.R.); cccaoye@163.com (Y.C.); 2 Jiangxi Health Industry Institute of Traditional Chinese Medicine, Nanchang 330115, China; 3 Tianjin Academy of Traditional Chinese Medicine, Tianjin 300120, China; 13156086136@163.com; 4 Institute of Medicinal Plant Development, Chinese Academy of Medical Sciences, Peking Union Medical College, Beijing 100193, China

**Keywords:** *Drynaria roosii*, sporophyll, proliferation, multi-omics, plant growth regulators

## Abstract

Sporophyte propagation in medicinal ferns is influenced by explant type, owing to their distinctive two-phase life cycle: haloid gametophyte and sporophyte. However, few studies have systematically investigated sporophyte proliferation using different explants. This study established an efficient in vitro propagation system for the medicinal fern *Drynaria roosii* using two types of explants: with green globules (A) and without them (B). An L_9_(3^4^) orthogonal design tested 6-benzylaminopurine (6-BA), 1-naphthaleneacetic acid (NAA), and 2,4-dichlorophenoxyacetic acid (2,4-D) on direct sporophyte regeneration. Results showed that sporophytes regenerated directly without a callus phase. For explant A, 6-BA was the dominant factor, with optimal proliferation at 3.00 mg/L 6-BA + 1.50 mg/L NAA + 0.05 mg/L 2,4-D. Explant B was less hormone-responsive but achieved a higher proliferation coefficient (116.50) under lower hormone levels. Endogenous hormone profiling revealed that increasing 6-BA triggered a biphasic cytokinin response, suppressed auxins, and elevated GA_9_ in explant A, while most hormones in explant B were significantly lower. Transcriptomic GO enrichment highlighted differentially expressed genes involved in the chloroplast envelope, thylakoid membrane, and photosynthesis, indicating that membrane remodeling and photosynthetic reorganization underpin sporophyte dedifferentiation and redifferentiation. We propose that 6-BA modulates proliferation through a biphasic cytokinin response, auxin suppression, and GA_9_ induction, coupled with transcriptomic reprogramming of the chloroplast envelope and thylakoid membrane components.

## 1. Introduction

Ferns are valuable plants in the world, widely used for ornamental, culinary and medicinal purposes [1,2,3]. In China, however, collection and utilization have relied almost exclusively on wild resources, which has led to a serious situation in which most ferns have become endangered—including all species of the genera *Cibotium* and *Ceratopteris*, which are listed in the National List of Key Protected Wild Plants. Thus, artificial cultivation has become increasingly important for the protection of wild fern resources. The sexual propagation by spores depends on the environmental conditions and seasonal variation, and can take a long time and easily encounter phytopathological problems [4]. Sporophyte breeding of ferns via tissue culture requires more effort and higher costs than that of seed plants, owing to the two independent life-history stages: the haploid gametophyte, which develops from spore germination, and the diploid sporophyte, which arises from the mature gametophyte [5,6]. Moreover, gametophyte proliferation under field conditions is limited, yielding a much smaller increase in biomass than that under sterile conditions, where gametophytes can undergo sustained propagation on suitable medium, as we observed in our preliminary experiments. Tissue culture is a highly effective protocol for rapid seedling production, avoiding seasonal constraints and land-use limitations. Moreover, stereoscopic planting patterns help resolve the conflict between the high cost of land management and the cultivation of medicinal plants. Amongst other factors, the selection of explants and combination of plant hormones determine the final seedling profiles.

*Drynaria roosii* (also named *Drynaria fortune* in early publications, referring to the same species) is the unique plant origin of Drynariae Rhizoma, the dried rhizomes of which are used for treating osteoporosis and traumatic fractures [1,7,8,9]. *D. roosii* is difficult to collect from the wild, as it is an epiphytic fern that often forms assemblages on tree trunks. There has been large-scale cultivation under forestry for production of the medicinal parts and supporting clinical and dairy usage in several provinces of China. Generating the juvenile sporophytes from direct spores is the most common method for industry production, which needs simple conditions and farming methods. However, we found that there was less proliferation of the gametophyte under the common cultivation conditions. Our primary research lasting three years indicated that the gametophyte stage of *D. roosii* can continue to proliferate after adding optimal medium and plant hormones under aseptic conditions. Presently, the induction and transformation of green globular bodies from gametophyte or sporophyte are common methods for obtaining mass sporophyte within a short time [10,11]. An interesting phenomenon is that the selection of explants influenced the induction efficiency: young sporophylls attached to green globular bodies (GGBs) derived from gametophytes produced fewer new sporophylls than those explants without green globular bodies [12]. We hypothesize that the variation and difference in endogenous plant hormone are the main factors leading to these differences in proliferation coefficient in sporophyte formation.

The generation and proliferation of sporophyte are regulated by phytohormone and internal function genes in the process of propagation in vitro. Generally, the combination of auxin and cytokinin formed an interaction framework in shoot branching and sporophyte proliferation [13]. Amongst these, abscisic acid played a crucial and lineage-specific role in the meristem specification of fern plants [14]. The auxin response participated in the developmental phase of fern because auxin transport was disrupted, which would lead to ectopic sporophyte induction on the fern gametophyte [15]. The published research has validated that genes of the Hairy Meristem family of the GRAS domain mainly maintained the totipotency of meristem in both growth stages: gametophytes and sporophytes [16]. Transcriptome sequencing and annotation analysis form a mature analytical technique for revealing the regulation mechanism of medicinal plants [17].

Here, we conducted a proliferation protocol using two types of explants combined with an orthogonal design. We then performed transcriptome analysis and targeted endogenous hormone metabolomics on selected groups to reveal the regulatory mechanisms underlying the differential proliferation between the two explant types. The comparison results provide a reference for plantlet production and the highly effective induction of sporophytes.

## 2. Results

### 2.1. Orthogonal Experimental Analysis and the Optimal Hormone Combination Scheme of D. roosii for Sporophyll Direct Organogenesis

**Figure 1:**
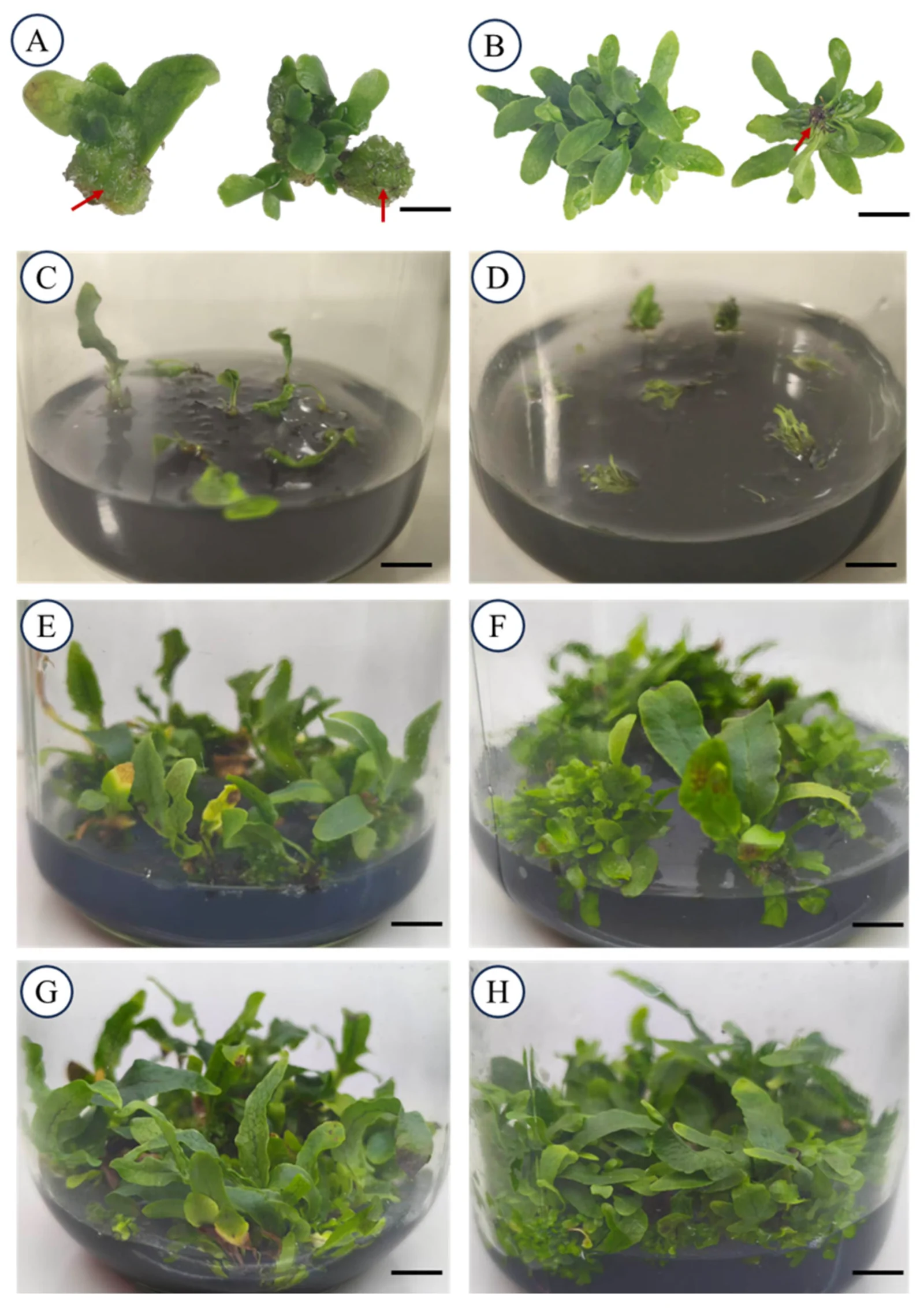
Morphological variation in sporophyte using two types of explants in *D. roosii*. (**A**) explant A containing globular body, bar = 0.33 cm; (**B**) explants B without globular body, bar = 0.88 cm; (**C**) explant A after initial culture, bar = 1.0 cm; (**D**) explant B after initial culture, bar = 1.0 cm; (**E**) explant A after 40-day culture, bar = 1.0 cm; (**F**) explant B after 40-day culture, bar = 1.0 cm; (**G**) explant A after 90-day initial culture, bar = 1.0 cm; (**H**) explant B after 90-day culture, bar = 1.0 cm). Note: Red arrows indicate the growth sites of green globular bodies (GGB).

The two types of explants (with GGB and without GGB; Figure 1A,B) were inoculated separately into Murashige and Skoog (MS) media with different hormone combinations. The initial explants with and without green globules are shown in Figure 1C and Figure 1D, respectively. After 40 days of culture, explant A produced 3–4 sporophylls per plantlet, with each sporophyte clearly distinguishable, whereas explant B generated a large number of clustered sporophylls (Figure 1E,F). Explant A exhibited more green globules on the sporophyll than explant B; however, explant B produced younger clustered sporophylls after a 90-day culture (Figure 1G,H). When these young sporophylls were subcultured individually, they showed a higher multiplication coefficient.

An orthogonal design L_9_(3^4^) was applied to evaluate the effects of three plant growth regulators (PGRs) (6-BA, NAA, and 2,4-D) on sporophyte proliferation using two types of explants (explant A and explant B). The range analysis results are summarized in Table 1. For explant A, the mean sporophyte amounts (k1, k2, k3) for 6-BA were 39.50, 60.33, and 73.22, respectively, with a range (R) of 33.72. For NAA, the k1–k3 values were 56.56, 53.39, and 63.11 (R = 9.72). For 2,4-D, the k1–k3 values were 53.67, 65.39, and 54.00 (R = 11.72). For explant B, the k1–k3 values for 6-BA were 91.17, 61.50, and 65.00 (R = 29.67); for NAA, they were 74.50, 63.50, and 79.67 (R = 16.17); for 2,4-D, they were 77.33, 68.83, and 71.50 (R = 8.50). For explant A, 6-BA had the greatest influence, followed by the 2,4-D and NAA. The optimal combination for explant A was 3.00 mg/L 6-BA + 1.50 mg/L NAA + 0.05 mg/L 2,4-D (A3B3C2), yielding a sporophyte amount of 76.00. For explant B, the optimal combination was 1.00 mg/L 6-BA + 1.50 mg/L NAA + 0.01 mg/L 2,4-D (A1B3C1).

Variance analysis of the orthogonal experiment was performed for explant A and explant B (Table 2). For explant A, 6-BA showed a significant effect on sporophyte proliferation (F = 6.4475, df = 2, *p* = 0.0320), while NAA (F = 0.3064, df = 2, *p* = 0.7470) and 2,4-D (F = 0.1979, df = 2, *p* = 0.8256) did not reach statistical significance. For explant B, none of the three hormones exhibited a significant effect: 6-BA (F = 1.4918, df = 2, *p* = 0.2979), NAA (F = 0.3280, df = 2, *p* = 0.7325), and 2,4-D (F = 0.0881, df = 2, *p* = 0.9168). Duncan’s multiple range test was conducted for the three levels of 6-BA on sporophyte proliferation in *D. roosii* (Table 3). It revealed a significant difference between 6-BA concentrations 1.00 and 3.00, whereas concentration 2.00 was not significantly different from either of the other two levels. The mean values for concentrations 1.00, 2.00, and 3.00 mg/L were 36.89, 59.17, and 73.22, respectively.

It should be noted that the optimal PGR combination (A1B3C1) identified by range analysis represents the best-performing treatment among the nine tested combinations in this orthogonal design. However, the ANOVA results indicated that the main effects of all three PGRs on explant B proliferation were not statistically significant (*p* > 0.05). Therefore, although this combination yielded the highest proliferation coefficient among the treatments, the differences among treatments were not statistically significant.

### 2.2. The Response Patterns of Endogenous Hormones in D. roosii Caused by 6-BA

After analyzing the individual factors in the orthogonal experiment, it was found that 6-BA had a significant effect on explant A. Therefore, we performed targeted endogenous hormone metabolomics analysis on groups 2, 5, and 8 to observe the response patterns of endogenous hormones in *D. roosii* under the influence of 6-BA. Compared to group 2, the hormone analysis of group 5 identified four differential expressed hormones (DEHs), including three upregulated cytokinins: 2-methylthio-cis-zeatin riboside, dihydrozeatin-O-glucoside riboside, and kinetin-9-glucoside; and one downregulated auxin(N-(3-indolylacetyl)-L-alanine), as shown in Table 4. For group 8, four DEHs were identified, including one cytokinin (N6-isopentenyladenosine) and two auxins (indole-3-acetic acid and N-(3-indolylacetyl)-L-alanine), all three of which were downregulated; and one upregulated gibberellin (gibberellin A9) (Table 5).

Meanwhile, to investigate the response patterns of endogenous hormones between the explants A and explant B, we simultaneously performed targeted hormone metabolomics analysis on the two explant groups of Group 1. Compared with explant A, a total of 16 DEHs was identified in explant B, including 5 downregulated auxins and 11 cytokinins. Among the cytokinins, two DEHs, meta-topolin and N6-benzyladenine-9-glucoside, were upregulated, while the remaining nine DEHs were downregulated (Table 6). Table 7 presents the standard curve equations, retention times, and determination coefficients (R^2^) for the significantly differential plant hormones identified by statistical testing, thereby strongly validating the absolute quantification of all the above-mentioned hormones.

### 2.3. Transcriptomic Analysis of D. roosii Impacted by 6-BA and Explant Selection

To investigate the role of 6-BA for differential induction performance in *D. roosii*, transcriptome analysis was performed on explant A treated with three gradient concentrations of 6-BA, as well as on explant A and explant B from the concentration combination of group 1. The sequencing results indicated that clean reads were mainly distributed between 43,054,865 and 57,854,177. Each sample yielded an average of 52,703,080.13 clean reads, with Q20 > 98.5% and Q30 > 93.12%. The average guanine and cytosine content (GC) was 47.74%. Overall data showed a high sequencing quality, suitable for in-depth investigation (Table 8).

**Figure 2:**
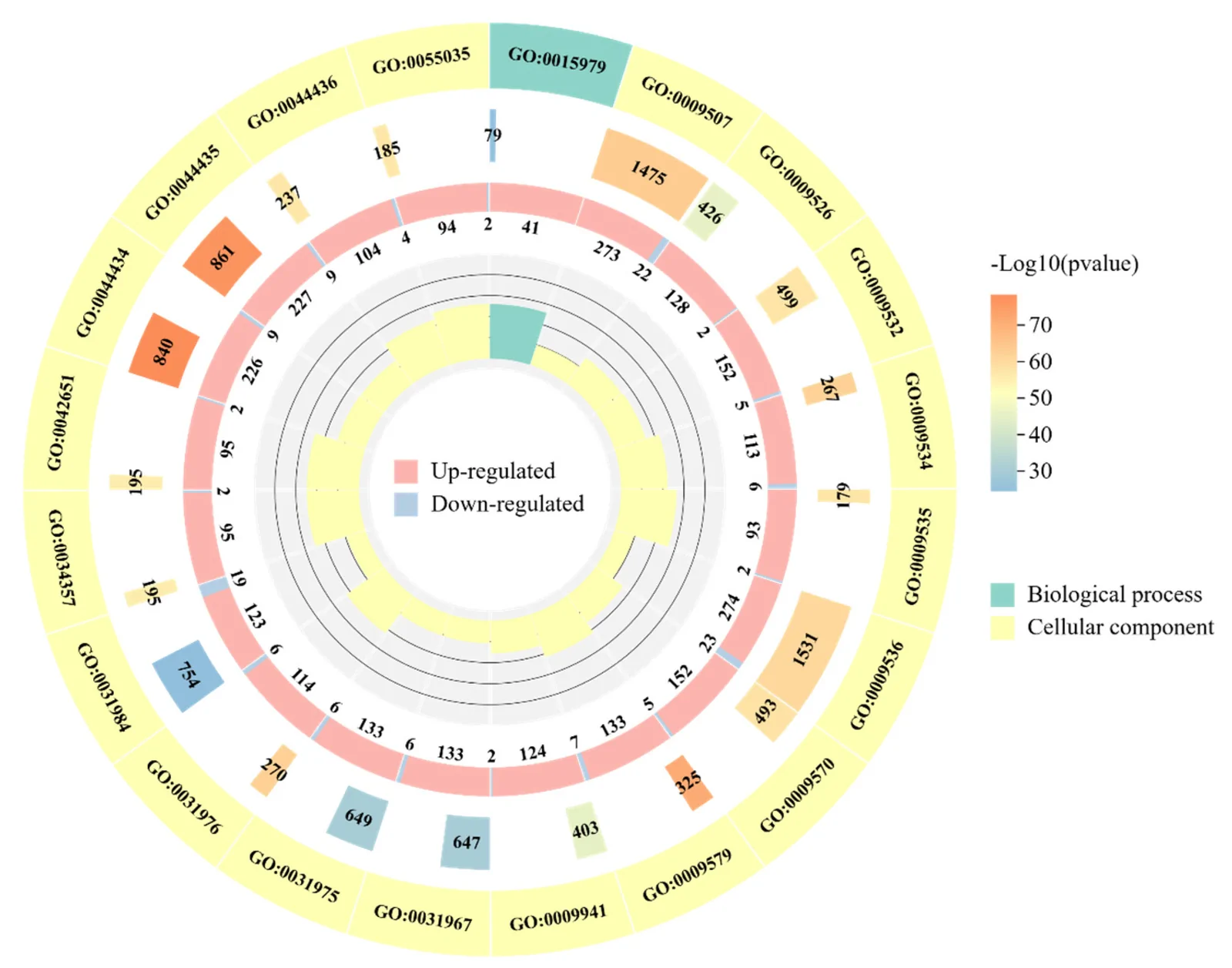
Circular plot of GO functional enrichment using DEGs of *D. roosii* derived from two explant types.

To visually compare the changes in gene functions under different concentrations of 6-BA treatments and between the two explant types (Figure 2), GO enrichment analysis was performed based on the functions of differentially expressed genes. The GO circular plot enrichment analysis revealed that in the ZD1 and ZB1 comparison, differentially expressed genes were significantly enriched in chloroplast/plastid-related structural and functional terms. At the biological process (BP) level, photosynthesis (GO:0015979) was the most prominently enriched term. At the cellular component (CC) level, the enriched terms exhibited a clear hierarchical distribution: the outer layer was enriched in plastid envelope (GO:0031975), chloroplast envelope (GO:0009941, GO:0009526), and plastid outer membrane (GO:0009527); extending inward, a large number of terms were enriched in thylakoid (GO:0009579), chloroplast thylakoid (GO:0009534), thylakoid membrane (GO:0042651), plastid thylakoid membrane (GO:0055035), and thylakoid components (GO:0044436, GO:0031984). In addition, chloroplast stroma (GO:0009570) was also significantly enriched. Compared with ZD1, ZB1 contained 2277 significantly differentially expressed genes, of which 1105 were upregulated and 1172 were downregulated (Figure S1A).

After differential gene screening, KEGG annotation was performed to identify the major pathways that were altered in *D. roosii* under different explant types and that ultimately led to differences in regeneration or developmental traits. Compared with ZD1, the significantly changed metabolic pathways in ZB1 mainly encompassed the following categories: signal transduction pathways (belonging to environmental information processing), translation, transcription, replication and repair (belonging to genetic information processing), carbohydrate metabolism, amino acid metabolism, lipid metabolism, energy metabolism, and biosynthesis of secondary metabolites (belonging to metabolism), cell growth and death, transport and catabolism (belonging to cellular processes), and ABC transporters (belonging to membrane transport), among others (Figure S1B).

**Figure 3:**
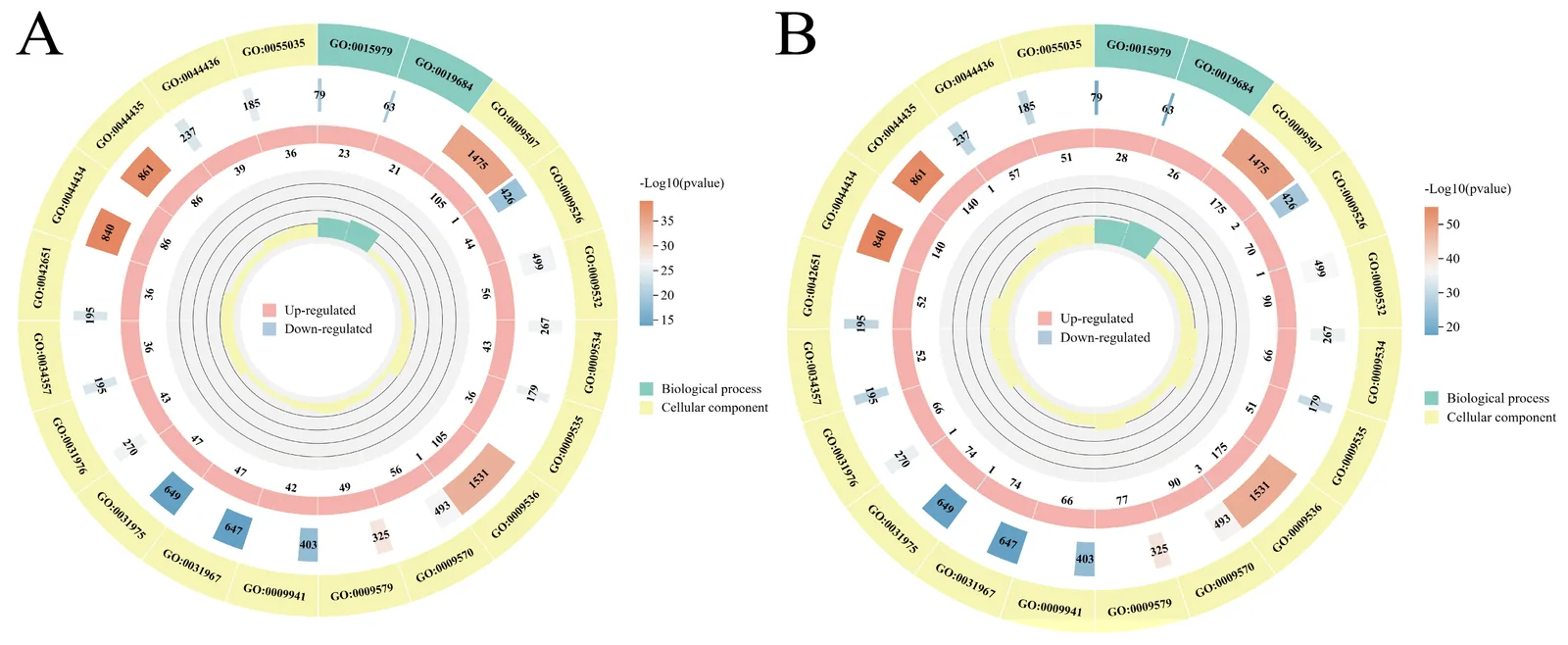
Circular plot of GO functional enrichment using DEGs of *D. roosii* derived from different concentrations of 6-BA. (**A**) Comparison between ZD2 and ZD5; (**B**) comparison between ZD2 and ZD8.

Compared with ZD2, the ZD5 and ZD8 groups exhibited highly similar enrichment patterns, both significantly enriched in biological processes such as photosynthesis (GO:0015979) and its light reaction (GO:0019684), as well as membrane structures including the thylakoid (GO:0009579), chloroplast thylakoid (GO:0009534), thylakoid membrane (GO:0042651), plastid thylakoid membrane (GO:0055035), and thylakoid component (GO:0044436). In addition, they also encompassed the chloroplast envelope (GO:0009941), plastid envelope (GO:0031975), chloroplast stroma (GO:0009570), and plastid (GO:0009536), showing a hierarchical enrichment pattern from the outer envelope to the inner thylakoid membranes. Only minor differences were observed between ZD5 and ZD8. The ZD5 group additionally enriched the photosynthetic membrane (GO:0034357) and plastid envelope (GO:0031976) (Figure 3A), whereas the ZD8 group additionally enriched the plastid stroma (GO:0009532) and repeatedly presented the photosystem II (GO:0009537) term (Figure 3B). Compared with ZD2, ZD5 had 396 upregulated and 56 downregulated genes, while ZD8 had 936 upregulated and 198 significantly downregulated genes (Figure S2A,B).

After differential gene screening, KEGG annotation was performed to identify the changes in *D. roosii* under gradient 6-BA concentrations. Integrating the annotation results from both ZD5 and ZD8 comparison groups revealed that, with changes in 6-BA concentration, the differentially expressed genes were commonly and significantly enriched in multiple core pathways. At the metabolic level, carbohydrate metabolism, energy metabolism, lipid metabolism, amino acid metabolism, and biosynthesis of secondary metabolites were all markedly altered, suggesting that 6-BA may influence cellular physiological status by modulating primary carbon/nitrogen metabolism and secondary product synthesis. At the genetic information processing level, translation, protein folding, and degradation pathways were significantly enriched, and signal transduction pathways as well as ABC transporter pathways were also regulated. In addition, concentration-specific differences were observed between the two groups: the ZD5 comparison showed more pronounced changes in terpenoid and polyketide metabolism, whereas ZD8 additionally enriched pathways related to nucleotide metabolism, transcriptional regulation, and cell motility. Overall, gradient 6-BA concentrations collectively mediated the concentration-dependent differences in regeneration capacity or secondary metabolite accumulation between different explant types of *D. roosii* through synergistic regulation of multiple processes, including signal perception, gene expression, energy supply, precursor biosynthesis, and transmembrane transport (Figure S2C,D).

## 3. Discussion

This study found that 6-BA is the critical factor influencing sporophyte proliferation in explant A (ZD1) of *D. roosii*, whereas proliferation in explant B (ZB1) was insensitive to the tested hormones through orthogonal experiments, suggesting fundamental differences in hormone homeostasis and differentiation potential between the two explant types. Meanwhile, as the primary factor, 6-BA influences the growth and development of *D. roosii* by affecting cell division and morphogenesis, which is consistent with previous findings in *Gardenia jasminoides* Ellis and ‘Tianhong 2’ Fuji nursery apple trees [18,19]. With increasing 6-BA concentrations, endogenous hormones exhibited a concentration-dependent response: the ZD5 group showed significant upregulation of multiple cytokinins, including dihydrozeatin-*O*-glucoside riboside and kinetin-9-glucoside, whereas the ZD8 group suppressed the accumulation of N^6^-isopentenyladenosine and IAA, while concurrently inducing the accumulation of gibberellin GA_9_. This low-concentration promotion, high-concentration inhibition hormonal response pattern may be associated with feedback regulation of endogenous hormone synthesis or glycosylation inactivation mediated by 6-BA, as has been documented in foliar application studies on apple and maize [20,21]. Notably, explant B exhibited a general downregulation of auxins and cytokinins compared with explant A (14 out of 16 differential hormones were downregulated), with particularly marked reductions in conjugated auxins such as IAA-Asp and IAA-Glu. We speculate that the lower hormone levels in explant B may relieve inhibition of cell division, thereby unleashing higher proliferative potential. In the construction of an efficient organogenesis system for *Suaeda glauca*, 6-BA exerted a significant effect in unleashing the proliferative potential of explants, confirming that the interplay between hormones and environmental factors critically determines regeneration efficiency [22]. This is a notion strongly supported by the orthogonal results, where the proliferation coefficient of explant B (116.5) far exceeded that of explant A (76.0), and this may be related to the PIN genes [23]. Furthermore, the upregulated metabolites meta-Topolin and N^6^-benzyladenine-9-glucoside in explant B may act as storage or transport forms of active cytokinins and may play a synergistic role in initiating clustered shoot differentiation.

Transcriptomic GO enrichment analysis further elucidated the molecular basis underlying the differences in proliferation. In the ZD1 and ZB1 comparison, differentially expressed genes were significantly enriched in the chloroplast envelope, thylakoid membrane, and photosynthesis-related terms, exhibiting a hierarchical distribution from the outer envelope to inner thylakoid membranes. This widespread differential expression of membrane-related genes suggests that explant B may be in a more active state of plastid differentiation or chloroplast biogenesis, and that membrane system remodeling is a key step in cell dedifferentiation and redifferentiation [19,24,25]. Notably, ZD5 and ZD8 groups also showed enrichment in the thylakoid membrane and photosynthesis terms compared with ZD2; however, unlike ZD1 and ZB1, their enrichment was more focused on the inner thylakoid membrane system, with ZD5 additionally enriching the “photosynthetic membrane” and ZD8 specifically enriching “photosystem II”. This difference may reflect differential regulation of chloroplast developmental stages by 6-BA concentration gradients: a low concentration (ZD5) promotes membrane system expansion, whereas a high concentration (ZD8) may accelerate photosystem assembly. Combining these findings with proliferation coefficients, we observed that groups with more active expression of membrane-related genes did not necessarily exhibit the highest proliferation rates—although explant B showed significant differences in membrane-related genes, its optimal proliferation occurred at low 6-BA (1.00 mg/L), whereas explant A proliferated best at moderate 6-BA (2.00 mg/L), where GO enrichment intensity fell between ZD2 and ZD8. This suggests that membrane system remodeling is a necessary but not sufficient condition for proliferation; proliferative efficiency depends on the synergistic balance between membrane system plasticity and endogenous hormone homeostasis.

Beyond hormone differences, explant choice, specifically the presence or absence of GGBs, may serve as an important determinant of sporophyte regeneration competence. We propose that attached GGBs might function as a developmental signaling hub, possibly maintaining adjacent sporophylls in a relatively quiescent state. This interpretation is consistent with the elevated conjugated auxins (IAA-Asp, IAA-Glu) and cytokinin storage forms observed in explant A—biochemical features commonly associated with growth suppression [26]. Thus, the higher exogenous 6-BA requirement (3.00 mg/L) for explant A might reflect the need to overcome this putative brake. By contrast, explant B (without GGB) exhibited generally lower endogenous hormone levels, which may lower the threshold for dedifferentiation [27]. This low endogenous hormone status may favor organogenesis by minimizing antagonistic cross-talk with exogenous PGRs, thereby achieving a higher proliferation coefficient (116.5) even under low hormone conditions. The transcriptomic enrichment of chloroplast envelope- and thylakoid membrane-related genes in explant B further supports this interpretation, likely reflecting active plastid structural remodeling—a process considered to be a hallmark of dedifferentiation prior to the acquisition of pluripotency [19]. Collectively, our findings suggest that regeneration outcomes are likely determined not by PGR concentrations alone, but rather by the interplay between exogenous hormones and the intrinsic developmental plasticity of the explants. Therefore, sporophylls without GGB may be more suitable for large-scale propagation, whereas explants containing GGB may serve as valuable models for investigating the molecular differences between gametophyte- and sporophyte-derived development.

**Figure 4:**
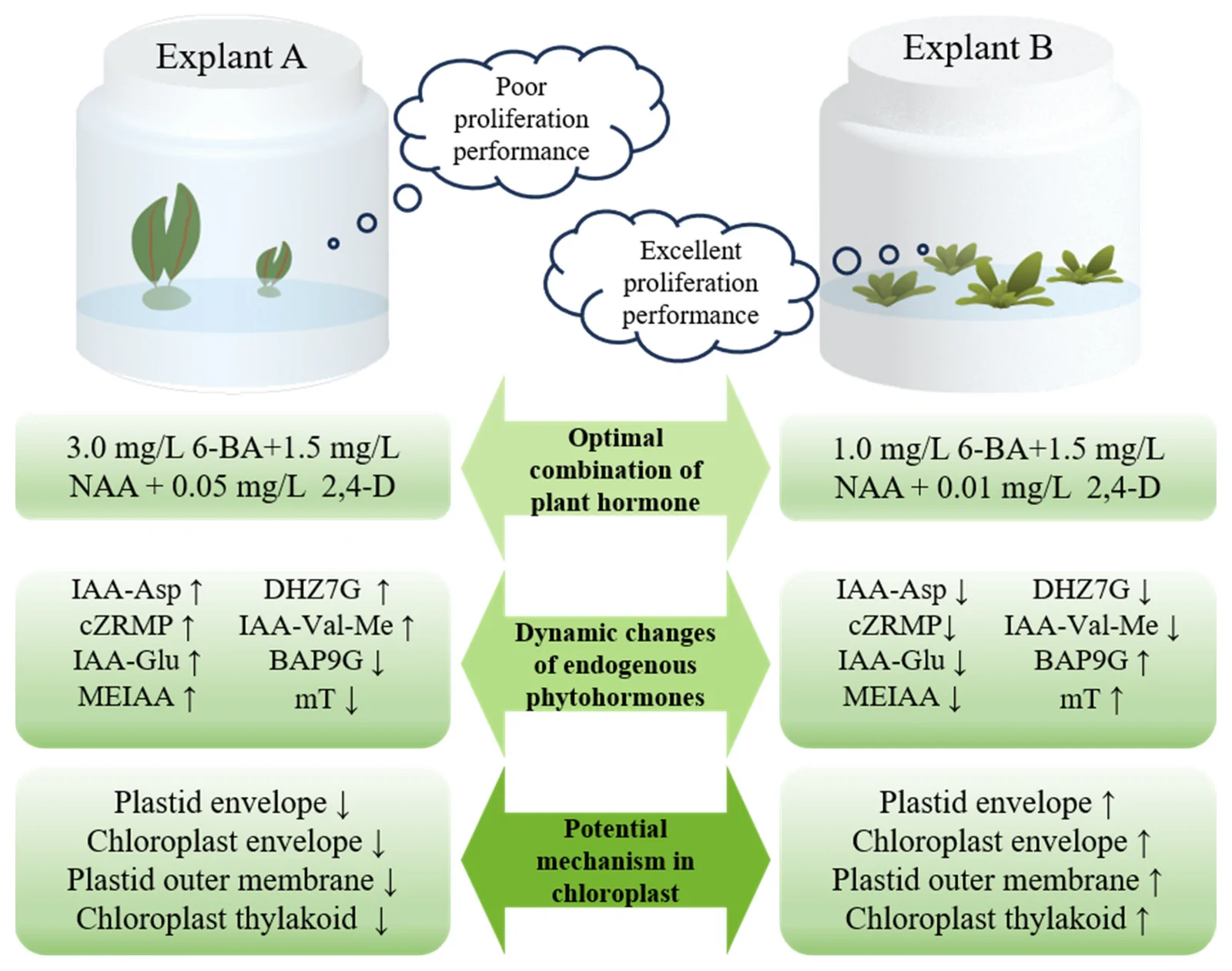
Schematic model illustrating the differential expression mechanisms between explant A (with green globules) and explant B (without green globules) in *D. roosii.* (IAA-Asp: Indole-3-acetyl-L-aspartic acid; cZRMP: cis-Zeatin riboside monophosphate; IAA-Glu: Indole-3-acetyl glutamic acid; MEIAA: Methyl indole-3-acetate; DHZ7G: Dihydrozeatin-7-glucoside; IAA-Val-Me: Indole-3-acetyl-L-valine methyl ester; BAP9G: N6-Benzyladenine-9-glucoside; mT: meta-Topolin; Upward arrows indicate up-regulation, while downward arrows indicate down-regulation.).

The auxin-to-cytokinin ratio has long been recognized as a pivotal regulatory switch that governs the commitment to callus formation, shoot organogenesis, or root differentiation in plant tissue culture [28,29,30]. We hypothesize that explant B, owing to its inherently lower endogenous hormone levels, is default-primed in a membrane-remodeled state more amenable to dedifferentiation, which may explain why exogenous hormones do not significantly promote its proliferation. In contrast, explant A requires exogenous 6-BA to disrupt its relatively stable membrane structure and initiate redifferentiation; however, excessively high 6-BA may limit proliferation efficiency by suppressing IAA and altering the membrane protein composition (Figure 4).

It should be noted that the orthogonal design (L_9_(3^4^)) employed in this study is a screening design. The primary objective was to rapidly screen for optimal proliferation formulations with a limited number of treatment combinations, rather than to comprehensively evaluate all main effects and interactions. Consequently, this design did not include zero-level controls (0 mg/L) for each plant growth regulator, nor could it provide a complete ANOVA for factor interactions (including the interaction between explant type and PGRs) or p-values for interaction terms. Nevertheless, this orthogonal screening strategy successfully provided efficient proliferation protocols that enabled our subsequent multi-omics mechanistic investigations. Future studies should build upon our current findings by employing full factorial designs with zero-level controls to comprehensively validate the main effects, interactions (including explant type × PGRs), and general robustness of the optimal combinations identified herein.

## 4. Materials and Methods

### 4.1. Materials and Experimental Design

Wild rhizomes of *D. roosii*, containing sporophyll, were collected from Huaqiao Country in Dexing City, Jiangxi Province, China (28.95° N, 117.76° E) and cultivated in the climate chamber at the Institute of Traditional Chinese Medicine Health Industry, China Academy of Chinese Medical Sciences from 2023, which was set at a temperature of 25 ± 2 °C, with a photoperiod of 8 h per day, relative air humidity of 55–70%, and light intensity of 2800 ± 300 lx. The mature spores generated from the newly sprouted sporophyll were collected, and we conducted a sterile generation system on a blank 1/2 MS medium. Two types of explants, sporophyte containing green globule (explant A, Figure 1A) and sporophyte without green globule at the bottom of the sporophyll (explant B, Figure 1B), were found on the induction medium of sporophyte after reaching the prothallus stage and sporophyte induction. Then, the two types of explants were subject to an orthogonal experimental design to find a suitable proliferation protocol of mass sporophytes.

### 4.2. Orthogonal Experimental Design for Sporophyte Proliferation of D. roosii

A screening experiment was performed using an L_9_(3^4^) orthogonal array to evaluate the effects of three plant growth regulators (6-BA, NAA, and 2,4-D) at different concentrations on sporophyte proliferation using two types of explants. The sporophytes with 2-3 sporophyll that had developed from prothalli were carefully removed, and the surrounding prothallus tissues were gently detached. An orthogonal experiment was then conducted using medium, adding different concentration of 6-benzylaminopurine (6-BA, 1, 2, 3 mg/L), 1-naphthaleneacetic acid (NAA, 0.5, 1, 1.5 mg/L), and 2,4-dichlorophenoxyacetic acid (2,4-D, 0.01, 0.05, 0.1 mg/L) (Table 1) using explant A and explant B, respectively. Five culture glass wide-mouth jars (300 mL, 6.5 cm in diameter and 9 cm in height) containing seven explants per bottle inoculated for each treatment group were maintained under conditions of 26 °C, 65% humidity, and 3000 lx irradiance. After 90 days of culture, the newly sprouted sporophytes were recorded for selection of the optimal medium, and the sporophyte proliferation coefficient was determined. The proliferation coefficient was calculated as the total number of separable and subculturable minimal propagule units (i.e., individual sporophyte plantlets capable of independent subculture) per culture vessel, divided by the initial number of explants inoculated per vessel (seven explants per jar). The basal medium consisted of MS basal salt mixture (Solarbio, Beijing, China) at a concentration of 4.74 g L^−1^, supplemented with 30 g L^−1^ sucrose as the carbon source, 1 g L^−1^ activated charcoal, and 4 g L^−1^ agar as a gelling agent. The pH of the medium was adjusted to 5.7–5.8 with 1 M NaOH or 1 M HCl prior to autoclaving at 121 °C for 20 min. The medium was dispensed into culture vessels at 50 mL per vessel, and the vessels were sealed with screw caps equipped with gas-permeable membranes.

### 4.3. Transcriptome Measurement of D. roosii

Total RNA was extracted from *D. roosii* samples previously stored at −80 °C using the RNAprep Pure Plant Kit (Beijing, China). RNA concentration and integrity were evaluated with an Agilent 2100 Bioanalyzer (Agilent Technologies, Santa Clara, CA, USA). Only high-quality RNA was subjected to mRNA enrichment using Oligo (dT) beads, followed by fragmentation and cDNA synthesis. The resulting cDNA was then ligated with poly-A tails and adapters to construct sequencing libraries, which underwent fragment size selection and PCR amplification. Library quality was confirmed by Q-PCR, with effective concentrations required to exceed 2 nM. In total, fifteen cDNA libraries were prepared and sequenced on the Illumina NovaSeq 6000 platform (Illumina, Inc., San Diego, CA, USA).

Raw reads were filtered to obtain clean reads by removing paired reads containing more than 10% unknown bases (N) or with over 50% of bases with a Phred quality score (Q) ≤ 20. The clean reads were assembled *de novo* into reference transcripts using Trinity (v2.4.0). After normalization of high-quality reads, gene expression levels were quantified as fragments per kilobase of transcript per million mapped reads (FPKM). Differentially expressed genes (DEGs) were identified using DESeq2 based on raw read counts, with screening criteria as a false discovery rate (FDR) < 0.05 and |log2Fold Change| ≥ 1. Functional annotation of DEGs was performed against the KEGG and the Gene Ontology (GO) databases to explore their potential involvement in cellular components, biological processes, and molecular functions, including roles in metabolite synthesis and other functional traits.

### 4.4. Measurement and Analysis of Targeted Hormone Metabolomics of D. roosii

A 50 mg aliquot of liquid nitrogen-ground tissue culture sample of *D. roosii* was weighed into a centrifuge tube. A 10 μL aliquot of an isotopic internal standard mixture (100 ng/mL) was added, followed by 1 mL of methanol/water/formic acid (15:4:1, *v*/*v*/*v*) as the extraction solvent. The mixture was vortexed for 10 min and then centrifuged at 12,000 rpm for 5 min at 4 °C. The supernatant was evaporated to dryness, and the residue was reconstituted in 100 μL of 80% methanol/water (*v*/*v*). The resulting solution was filtered through a 0.22 μm membrane filter and transferred into an autosampler vial for LC-MS/MS analysis.

Chromatographic separation was performed on a Waters ACQUITY UPLC HSS T3 C18 column (Waters Corporation, Milford, MA, USA, 1.8 μm, 100 mm × 2.1 mm) maintained at 40 °C, with a flow rate of 0.35 mL/min and an injection volume of 2 μL. Mobile phase A was ultrapure water, and mobile phase B was acetonitrile, both of which contained 0.04% acetic acid. The gradient elution program was 0–1 min, 5% B; 1–8 min, 5% → 95% B; 8–9 min, 95% B; 9.1–12 min, 5% B.

Mass spectrometry was performed using an AB SCIEX QTRAP 6500 mass spectrometer (AB Sciex, Framingham, MA, USA) equipped with an electrospray ionization (ESI) source operating in polarity-switching positive/negative ion mode. The ion source temperature was set as 550 °C, ion spray voltage was 5500 V under the positive mode and −4500 V under the negative mode, and curtain gas was 35 psi. Data were acquired in the multiple reaction monitoring (MRM) mode, and each ion transition was detected using the optimized declustering potential (DP) and collision energy (CE). Metabolites were identified by comparing retention times and MRM ion pair information against an in-house database constructed from authentic standards.

Quantification was performed using the isotope internal standard method. A series of standard solutions at concentration gradients (0.01–500 ng/mL, with the range appropriately extended for certain compounds) was prepared. Calibration curves were constructed by plotting the concentration ratio of the analyte to the internal standard (X) against the peak area ratio (Y). The peak area ratio of the analyte in the sample was substituted into the calibration curve to obtain the calculated concentration. The actual content (ng/g) was then calculated using the following formula:

Content (ng/g) = (c × V1/1000/V2) × V3/m

c = concentration calculated from the calibration curve (ng/mL), V1 = reconstitution volume (μL), V2 = aliquot volume taken for analysis (μL), V3 = extraction solvent volume (μL), m = sample mass (g).

### 4.5. Statistical Analysis

Each experiment was performed with three independent biological replicates, and the results are presented as the mean ± standard deviation (SD). Significant differences were determined using Student’s *t*-test. For comparisons among multiple groups, one-way analysis of variance (ANOVA) was performed, followed by Duncan’s multiple range test, with different lowercase letters used to denote significant differences at *p* < 0.05. Multi-omics analysis in this study was conducted using the Omicsmart interactive platform (www.omicsmart.com). Gene Ontology (GO) enrichment circular plotting was performed using the Metware online platform (https://www.metware.cn).

## 5. Conclusions

This study used two types of explants of *D. roosii* to investigate direct sporophyte regeneration via an orthogonal design combined with targeted hormone metabolomics and transcriptome sequencing. Results showed that sporophytes could arise directly from explants without an intervening callus stage. 6-BA was the primary factor affecting proliferation in explants containing green globules, with an optimal concentration of 3.00 mg/L. Explants without green globules were insensitive to exogenous hormones but exhibited higher intrinsic proliferative capacity. Endogenous hormone profiling revealed that increasing 6-BA concentrations caused a shift from increased to decreased cytokinins, suppressed auxins, and induced gibberellins, with general downregulation of auxins and cytokinins in explants without green globules. GO enrichment of transcriptomic data showed significant enrichment in the chloroplast envelope, thylakoid membrane, and photosynthesis-related terms, suggesting that membrane remodeling and photosynthetic structure reorganization are critical molecular events associated with dedifferentiation and proliferation efficiency. Collectively, these findings provide a theoretical basis for efficient in vitro propagation of *D. roosii*.

## Figures and Tables

**Table 1:** Experimental design and comparison results of L_9_(3^4^) orthogonal approach for sporophyte proliferation in *D. roosii*.

Codes		Concentration of Plant Growth Regulators (mg/L)				Sporophyte Amount	
		6-BA	NAA	2,4-D	Error	Explant A	Explant B
1		1.00	0.50	0.01	(1)	24.00	99.00
2		1.00	1.00	0.05	(2)	50.50	58.00
3		1.00	1.50	0.10	(3)	44.00	116.50
4		2.00	0.50	0.05	(3)	69.67	79.00
5		2.00	1.00	0.10	(1)	42.00	52.50
6		2.00	1.50	0.01	(2)	69.33	53.00
7		3.00	0.50	0.10	(2)	76.00	45.50
8		3.00	1.00	0.01	(3)	67.67	80.00
9		3.00	1.50	0.05	(1)	76.00	69.50
Sporophyte amount (Explant A)	K_1_	39.50	56.56	53.67	47.33		
	K_2_	60.33	53.39	65.39	65.28		
	K_3_	73.22	63.11	54.00	60.45		
	R_2_	33.72	9.72	11.72	17.95		
Sporophyte amount (Explant B)	K_1_	91.17	74.50	77.33	73.67		
	K_2_	61.50	63.50	68.83	52.17		
	K_3_	65.00	79.67	71.50	91.83		
	R_2_	29.67	16.17	8.50	39.67		

Notes: Explant A: sporophytes with green globular bodies (GGBs); Explant B: sporophytes without GGBs. 6-BA: 6-benzylaminopurine; NAA: 1-naphthaleneacetic acid; 2,4-D: 2,4-dichlorophenoxyacetic acid. K_1_, K_2_, and K_3_ represent the mean sporophyte amounts at the three levels (1.00, 2.00, 3.00 mg/L for 6-BA; 0.50, 1.00, 1.50 mg/L for NAA; 0.01, 0.05, 0.10 mg/L for 2,4-D) of each factor. R denotes the range (max K–min K) for each factor, indicating the relative strength of its effect. Error column represents the blank (dummy) factor used for variance estimation in the orthogonal design. Data are presented as mean values (n Five replicate jars per treatment, and per jar contained 7 explants).

**Table 2:** Variance analysis results of orthogonal approach for sporophyte proliferation in *D. roosii*.

Variables		Sum of Type III Square	Degree of Freedom	Mean Square	F Value	*p* Value
Explant A	6-BA	2013.9691	2.0000	1006.9846	6.4475	0.0320
	NAA	273.4506	2.0000	136.7253	0.3064	0.7470
	2,4-D	182.5988	2.0000	91.2994	0.1979	0.8256
Explant B	6-BA	1503.5000	2.0000	751.7500	1.4918	0.2979
	NAA	446.1667	2.0000	223.0833	0.3280	0.7325
	2,4-D	129.1667	2.0000	64.5833	0.0881	0.9168

Notes: Factors with *p* < 0.05 were considered statistically significant.

**Table 3:** Duncan test under three levels of 6-BA for sporophyte proliferation in *D. roosii*.

Concentration	Averaged Value	Significance Label
1	36.89	a
2	59.17	ab
3	73.22	b

Notes: Values represent the mean sporophyte amounts at each 6-BA concentration. Different lowercase letters indicate significant differences at *p* < 0.05 as determined by Duncan’s multiple range test.

**Table 4:** Content comparison of plant hormones between ZD2 and ZD5 groups in *D. roosii*.

Plant Hormones	Categories	ZD2	ZD5	*p*-Value	FDR	FC	Type
2-Methylthio-cis-zeatin riboside	Cytokinin	0.19 ± 0.09	0.53 ± 0.19	0.11	0.65	2.74	up
Dihydrozeatin-O-glucoside riboside	Cytokinin	0.02 ± 0.03	0.09 ± 0.02	0.05	0.59	4.51	up
Kinetin-9-glucoside	Cytokinin	0.19 ± 0.27	1.54 ± 0.84	0.14	0.65	8.05	up
N-(3-indolylacetyl)-L-alanine	Auxin	0.56 ± 0.39	0	0.18	0.65	0.00	down

**Table 5:** Content comparison of plant hormones between ZD2 and ZD8 groups in *D. roosii*.

Plant Hormones	Categories	ZD2	ZD8	*p*-Value	FDR	FC	Type
N6-isopentenyladenosine	Cytokinin	0.20 ± 0.16	0.10 ± 0.01	0.44	0.92	0.47	down
Indole-3-acetic acid	Auxin	2.48 ± 0.41	0.62 ± 0.88	0.08	0.92	0.25	down
N-(3-Indolylacetyl)-L-alanine	Auxin	0.56 ± 0.39	0	0.18	0.92	0	down
GA_9_	GA	0	1.76 ± 1.3	0.20	0.92	NA	up

**Table 6:** Content comparison of plant hormones between ZB1 and ZD1 groups in *D. roosii*.

Plant Hormones	Categories	ZD1	ZB1	*p*-Value	FDR	FC	Type
Indole-3-acetyl glutamic acid	Auxin	3.30 ± 1.07	1.05 ± 0.04	0.10	0.41	0.32	down
Indole-3-acetyl-L-aspartic acid	Auxin	40.74 ± 4.26	11.43 ± 4.63	0.00	0.09	0.28	down
Indole-3-acetyl-L-valine methyl ester	Auxin	0.05 ± 0.05	0.01 ± 0.01	0.42	0.61	0.20	down
Methyl indole-3-acetate	Auxin	0.27 ± 0.14	0.13 ± 0.02	0.28	0.51	0.48	down
N-(3-indolylacetyl)-L-valine	Auxin	1.34 ± 0.55	0.55 ± 0.19	0.17	0.51	0.41	down
2-Methylthio-cis-zeatin	Cytokinin	1.13 ± 0.21	0.50 ± 0.36	0.12	0.41	0.44	down
2-Methylthio-cis-zeatin riboside	Cytokinin	0.31 ± 0.19	0.12 ± 0.05	0.28	0.51	0.38	down
cis-Zeatin riboside	Cytokinin	2.64 ± 1.05	1.28 ± 0.73	0.22	0.51	0.49	down
cis-Zeatin riboside monophosphate	Cytokinin	1.90 ± 0.35	0.94 ± 0.04	0.06	0.41	0.50	down
cis-Zeatin-9-glucoside	Cytokinin	0.81 ± 0.58	0.22 ± 0.16	0.28	0.51	0.27	down
cis-Zeatin-O-glucoside riboside	Cytokinin	11.79 ± 3.04	5.59 ± 2.65	0.10	0.41	0.47	down
Dihydrozeatin-7-glucoside	Cytokinin	0.20 ± 0.14	0.03 ± 0.04	0.22	0.51	0.14	down
meta-Topolin	Cytokinin	0.02 ± 0.02	0.03 ± 0.02	0.50	0.70	2.09	up
N6-Benzyladenine-9-glucoside	Cytokinin	0.15 ± 0.11	0.41 ± 0.34	0.40	0.61	2.73	up
N6-isopentenyladenosine	Cytokinin	0.37 ± 0.23	0.06 ± 0.02	0.20	0.51	0.15	down
N-6-iso-pentenyladenosine-5′-monophosphate	Cytokinin	2.80 ± 0.61	0.69 ± 0.09	0.04	0.40	0.25	down

**Table 7:** Linear equation of plant hormones with significant difference.

Plant Hormones	Retention Time	Equation	R2
2-Methylthio-cis-zeatin	4.69	y = 0.02481 x − 3.50312 × 10^−5^	0.9921
2-Methylthio-cis-zeatin riboside	4.76	y = 0.11987 x + 7.50771 × 10^−4^	0.99421
cis-Zeatin riboside	3.80	y = 0.15988 x − 1.36788 × 10^−4^	0.99248
cis-Zeatin riboside monophosphate	3.15	y = 0.01817 x − 9.85256 × 10^−4^	0.99167
cis-Zeatin-9-glucoside	3.39	y = 0.42458 x + 1.58860 × 10^−4^	0.99056
cis-Zeatin-O-glucoside riboside	3.62	y = 0.05315 x − 9.01769 × 10^−4^	0.99
Dihydrozeatin-7-glucoside	3.29	y = 0.35849 x − 1.12962 × 10^−4^	0.99372
Dihydrozeatin-O-glucoside riboside	3.59	y = 0.31493 x − 0.00135	0.99956
Gibberellin A9	7.18	y = 0.06645 x + 0.00393	0.99548
Indole-3-acetic acid	5.26	y = 0.01316 x − 9.59030 × 10^−4^	0.99981
Indole-3-acetyl glutamic acid	4.56	y = 0.12701 x − 8.01864 × 10^−4^	0.99059
Indole-3-acetyl-L-aspartic acid	4.43	y = 0.02135 x − 0.00402	0.99063
Indole-3-acetyl-L-valine methyl ester	6.43	y = 1.63815 x + 0.03459	0.99275
Kinetin-9-glucoside	3.93	y = 0.22614 x + 9.74597 × 10^−5^	0.99001
meta-Topolin	3.86	y = 0.09785 x + 3.86888 × 10^−5^	0.99125
Methyl indole-3-acetate	6.41	y = 0.06898 x − 2.97137 × 10^−4^	0.99187
N-(3-indolylacetyl)-L-alanine	4.91	y = 0.19467 x − 0.01654	0.99971
N-(3-indolylacetyl)-L-valine	5.64	y = 0.57931 x + 0.00232	0.9908
N6-Benzyladenine-9-glucoside	4.41	y = 0.33719 x + 0.00140	0.99291
N6-isopentenyladenosine	4.27	y = 0.05423 x + 1.25977 × 10^−4^	0.99704
N-6-iso-pentenyladenosine-5′-monophosphate	3.91	y = 0.04485 x − 3.69939 × 10^−4^	0.99622

**Table 8:** Sequencing quality of all samples in *D. roosii*.

Sample	Raw Reads	Clean Reads	Clean Ratio	Q20	Q30	GC
ZB1	43,195,915.3333	43,054,864.6667	0.9967	0.9850	0.9312	0.4768
ZD1	58,019,777.3333	57,854,177.3333	0.9972	0.9894	0.9442	0.4815
ZD2	56,524,174.6667	56,347,784.0000	0.9970	0.9859	0.9357	0.4771
ZD5	55,454,763.3333	55,241,296.0000	0.9965	0.9864	0.9381	0.4764
ZD8	51,197,046.0000	51,017,278.6667	0.9966	0.9851	0.9335	0.4754

Note: Q20: proportion of bases with a sequencing error rate < 1%; Q30: proportion of bases with a sequencing error rate < 0.1%; GC content: percentage of G and C bases among total bases.

## Data Availability

Dataset available on request from the authors, and the raw data supporting the conclusions of this article will be made available by the authors on request.

## Supplementary Materials

The following supporting information can be downloaded at https://www.mdpi.com/article/10.3390/plants15162543/s1, Figure S1: Differential expression analysis and KEGG functional classification of DEGs between ZD1 and ZB1 combinations. (A) Volcano plot of differentially expressed genes (DEGs) between group ZD1 and group ZB1, (B) KEGG enrichment classification histogram of DEGs in the ZD1 and ZB1 comparison groups; Figure S2: Differential expression analysis and KEGG functional classification of DEGs between ZD2, ZD5 and ZD8 combinations. (A) Volcano plot of differentially expressed genes (DEGs) between group ZD1 and group ZD5, (B) volcano plot of differentially expressed genes (DEGs) between group ZD1 and group ZD8, (C) KEGG enrichment classification histogram of DEGs in the ZD2 and ZD5 comparison groups, (D) KEGG enrichment classification histogram of DEGs in the ZD2 and ZD8 comparison groups.

## Abbreviations

The following abbreviations are used in this manuscript:

GGB	Green globular body
MS	Murashige and Skoog
6-BA	6-benzylaminopurine
NAA	1-naphthaleneacetic acid
2,4-D	2,4-dichlorophenoxyacetic acid
N	Unknown bases
DEGs	Differentially expressed genes
FDR	False discovery rate
GO	Gene Ontology
ESI	Electrospray ionization
MRM	Multiple reaction monitoring
DP	Declustering potential
CE	Collision energy
SD	Standard deviation
ANOVA	One-way analysis of variance
R^2^	Determination coefficients
GC	Guanine and cytosine content
BP	Biological process
CC	Cellular component
FPKM	Fragments per kilobase of transcript per million mapped reads
PGR	Plant growth regulator
